# Correction: Discovery of 8-prenylnaringenin from hop (*Humulus lupulus* L.) as a potent monoacylglycerol lipase inhibitor for treatments of neuroinflammation and Alzheimer’s disease

**DOI:** 10.1039/d2ra90070j

**Published:** 2022-07-13

**Authors:** Min-Che Tung, Kit-Man Fung, Hsing-Mien Hsu, Tien-Sheng Tseng

**Affiliations:** Division of Urology, Department of Surgery Tungs’ Taichung MetroHarbor Hospital Taichung 435 Taiwan; Institute of Biological Chemistry, Academia Sinica Taipei 115 Taiwan; Institute of Molecular Biology, National Chung Hsing University Taichung Taiwan emersontseng@dragon.nchu.edu.tw

## Abstract

Correction for ‘Discovery of 8-prenylnaringenin from hop (*Humulus lupulus* L.) as a potent monoacylglycerol lipase inhibitor for treatments of neuroinflammation and Alzheimer’s disease’ by Min-Che Tung *et al.*, *RSC Adv.*, 2021, **11**, 31062–31072, https://doi.org/10.1039/D1RA05311F.

The authors regret that the name of one of the authors (Hsing-Mien Hsu) was shown incorrectly in the original article. The corrected author list is as shown above.

The authors also regret an incorrect version of Fig. 7 was included in the original article. The correct version of Fig. 7 is presented below.

**Fig. 7:**
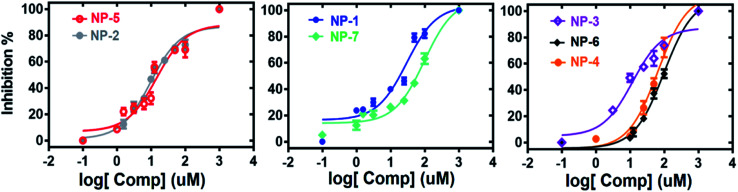
The dose-dependent inhibitions of the identified inhibitors against hMAGL.

The Royal Society of Chemistry apologises for these errors and any consequent inconvenience to authors and readers.

## Supplementary Material

